# On Glaucoma

**Published:** 1830-10-01

**Authors:** 


### XXVI.

### On Glaucoma. By William Mackenzie, Lecturer on the Eye in the University of Glasgow, &c.

This zealous cultivator of ophthalmology has published a paper on the above subject in the eleventh number of our Glasgow cotemporary, of which we shall offer an analysis to our readers. We shall begin with the pathological anatomy. Very little on this subject is recorded even by the best ophthalmologists. Mr. M. was anxious for an opportunity of dissecting some glaucomatous eyes, and lately was favoured with several in that state. The following are the particulars which he observed.

"1. The choroid coat, and especially the portion of it in contact with the retina, of a light brown colour, without any appearance of pigmentum nigrum.

2. The vitreous humour in a fluid state; perfectly pellucid; colourless, or slightly yellow. No trace of hyaloid membrane.

3. The lens of a yellow or amber colour, especially towards its centre; its consistence firm; and its transparency perfect, or nearly so.

4. In the retina, no trace of limbus luteus, or foramen centrale.

To the first of these changes, namely, the deficiency of pigmentum nigrum, I am inclined to ascribe, in a great measure, the opaque appearance of the deep-seated parts of the eye in glaucoma. This appearance I regard as a reflection merely of the light from the retina, choroid, and sclerotica; it is probably bluish when it first leaves the reflecting surface formed by these membranes, but immediately assumes a greenish hue from passing through the yellowish fluid which occupies the place of the vitreous humour, and through the lens, which is still more decidedly of a yellow, or even amber colour, at that period of life when glaucoma is most apt to attack the eye.

Scarpa has adopted a similar view of the nature of Glaucoma, namely, that it is a reflection; but he assumes, seemingly without proof, that it is from a thickened retina that the reflection takes place. After mentioning that those cases of amaurosis may be regarded as incurable, in which the bottom of the eye presents an unusual paleness, similar to horn, sometimes inclining to green, and reflected from the retina as if from a mirror, he adds, in a note, the following remarks. 'The retina of a sound eye is transparent; and, therefore, in whatever degree of dilatation the pupil may be, the bottom of the eye is of a deep black. That unusual paleness, then, which accompanies amaurosis, indicates that a remarkable change has happened in the substance of the optic nerve forming the retina, which, according to all appearance, is become thickened, and rendered permanently incapable of transmitting the impressions of light.'"

Mr. M. has never detected any other change in the retina than what is above-mentioned—namely, a want of the limbus luteus and foramen centrale.

*Symptomatology.* Limited and sluggish motion of the pupil, with other amaurotic symptoms, always attend glaucoma. Ultimately the pupil is dilated, and the retina becomes insensible to light. The loss of sight is generally gradual. The want of pigmentum nigrum may sufficiently explain the weakness of sight which accompanies glaucoma in the early stages. The pressure of the accumulated fluid within the eye is probably the cause of the total blindness which results at last.

"If the pupil of a glaucomatous eye is small, the appearances are apt to impose on the inexperienced observer for those of cataract. The colour, however, of the glaucomatous eye, is sufficient to prove that the case is at any rate not one of simple lenticular cataract, for opacity of the lens alone is never green. A green cataract is always attended with glaucoma. On dilating the pupil by belladonna, the green appearance presented in simple glaucoma seems to retire to a greater depth behind the iris, and becomes more circumscribed.

Glaucoma is frequently combined with arthritic inflammation. When this is the case the sclerotica and conjunctiva become loaded with varicose vessels of a livid colour, the pupil dilates irregularly, the lens becomes opaque, and is pushed forward so as almost to touch the cornea; the junction of the sclerotica and cornea becomes of a pearly-white colour; racking pain is complained of in the eye and head, and vision becomes totally extinct. After some time, the inflammatory symptoms subside, and the contents of the eyeball begin to be absorbed, so that it shrinks to less than its natural size, and, instead of the preternatural hardness which it formerly presented, becomes boggy.

The symptoms which we gather from the testimony of the patient, are the following:—vix. sensations of fiery and prismatic spectra, muscæ volitantes, misty and indistinct vision, and pain across the forehead, which is, at first slight, but often becomes severe. Not unfrequently those who become affected with glaucoma have long suffered from those pains in the teeth and head, which are generally accounted rheumatic. In some instances the glaucomatous eye is still sensible to objects placed to one or other side of the patient, while in every other direction it distinguishes nothing."

*Proximate cause.* Mr. M. thinks that inflammation may be the cause which leads to the destruction of the hyaloid membrane; and that this, in its turn, may produce a series of other changes. It is also probable, he observes, that the superabundance of the aqueous humour, promotes by pressure, the absorption of pigmentum nigrum, rendering the retina insensible in the end. The destruction of the hyaloid membrane and superabundance of fluid afterwards occupying the place of the vitreous humour, are looked upon by our author as the essential changes which take place in glaucoma.

*Exciting and Predisposing Causes.* The Germans consider glaucoma as almost always connected with arthritis, or rather as the result of chronic arthritic inflammation. It is much more frequently met with in old than in young subjects—rarely occurring before the age of 40—frequently after 60. Mr. M. has been led to suspect that the habitual use of spirits and tobacco operates in the production of this disease.

*Prognosis.* When glaucoma has commenced in one eye, it generally extends to the other. In its fully formed state, it is absolutely incurable. But it may often be checked in its progress; and, when only one eye is affected, it may sometimes be prevented from extending to the other. We cannot restore the secretion of pigmentum nigrum, but remedies may occasionally arrest the disease, and even improve the impaired vision.

*Treatment.* This we shall give in the concise language of the talented author.

"1. On the presumption that glaucoma originates in an inflammatory affection of the hyaloid membrane, bleeding and purging have been employed in order to arrest its progress; and occasionally this practice has been attended with benefit. Counter-irritation, also, has been found useful, and especially the tartar emetic eruption between the shoulders.

2. Calomel, with opium, has been given, on the principle that in almost all cases of deep-seated inflammation of the eye, mercury proves salutary. As is the case in arthritic ophthalmia, with which glaucoma is certainly allied, an alterative course will prove more beneficial than if the mercury were pushed so as severely to affect the mouth. Indeed, it is evident that from the age and constitution of those who are in general the subjects of glaucoma, neither depletion nor mercurialization can, with propriety, be employed, without more than ordinary caution.

3. Rest of the eyes, a mild diet, a healthy state of the skin, and abstinence from alcoholic fluids, and tobacco in every form, must be enjoined.

4. Arthritic inflammation of the eye is often greatly benefited by the use of tonics; as precipitated carbonate of iron, sulphate of quina, and the like. After depletion, such remedies may be also tried in glaucoma.

5. Dilatation of the pupil by belladonna greatly improves the vision of most glaucomatous eyes, and may be employed day after day as a palliative. The most convenient mode of applying the belladonna is in aqueous solution, filtered through paper, and dropped upon the conjunctiva morning and evening.

6. As a superabundance of dissolved vitreous humour appears to form an essential part of the morbid changes which take place in the glaucomatous eye, it is not unreasonable to conclude that occasionally puncturing the sclerotica and choroid might prove serviceable, by relieving the pressure of the accumulated fluid on the retina. The puncture should be made with a broad iris-knife, at the usual place of entering the needle in the operation of couching. The instrument should be pushed towards the centre of the vitreous humour, turned a little on its axis, and held for a minute or two in the same position, so that the fluid may be allowed to escape.

7. The removal of the crystalline lens from a glaucomatous eye not only lessens very much the greenish appearance of the humours, but improves the vision of the patient. At the same time, although I am persuaded that the absence of the lens might be advantageous even in the early stage of this disease, and prevent, in a considerable measure, its further progress, extraction is an operation, which I would by no means venture to recommend for general adoption in such cases. The patient generally sees too much to warrant our exposing him to the danger of arthritic inflammation coming on after the operation. I have known glaucoma operated on for cataract; that is to say, the amber-coloured lens removed by extraction, the operator apprehending that he was removing an opaque or cataractous lens; and I have seen the incision, after such an operation, heal without inflammation, and the patient receive a considerable accession of vision. But I have also known such violent inflammation follow the removal of the lens from a glaucomatous eye, as entirely destroyed the natural structure of the most important parts of the organ."

---

